# Differences in Lateral Line Morphology between Hatchery- and Wild-Origin Steelhead

**DOI:** 10.1371/journal.pone.0059162

**Published:** 2013-03-15

**Authors:** Andrew D. Brown, Joseph A. Sisneros, Tyler Jurasin, Chau Nguyen, Allison B. Coffin

**Affiliations:** 1 Department of Speech & Hearing Sciences, University of Washington, Seattle, Washington, United States of America; 2 Departments of Psychology and Biology, University of Washington, Seattle, Washington, United States of America; 3 Department of Fisheries, Quinault Indian Nation, Taholah, Washington, United States of America; 4 College of Arts and Sciences, Washington State University, Vancouver, Washington, United States of America; 5 Department of Veterinary and Comparative Anatomy, Pharmacology, and Physiology, Washington State University, Vancouver, Washington, United States of America; Universität Bielefeld, Germany

## Abstract

Despite identification of multiple factors mediating salmon survival, significant disparities in survival-to-adulthood among hatchery- versus wild-origin juveniles persist. In the present report, we explore the hypothesis that hatchery-reared juveniles might exhibit morphological defects in vulnerable mechanosensory systems prior to release from the hatchery, potentiating reduced survival after release. Juvenile steelhead (*Oncorhynchus mykiss*) from two different hatcheries were compared to wild-origin juveniles on several morphological traits including lateral line structure, otolith composition (a proxy for auditory function), and brain weight. Wild juveniles were found to possess significantly more superficial lateral line neuromasts than hatchery-reared juveniles, although the number of hair cells within individual neuromasts was not significantly different across groups. Wild juveniles were also found to possess primarily normal, aragonite-containing otoliths, while hatchery-reared juveniles possessed a high proportion of crystallized (vaterite) otoliths. Finally, wild juveniles were found to have significantly larger brains than hatchery-reared juveniles. These differences together predict reduced sensitivity to biologically important hydrodynamic and acoustic signals from natural biotic (predator, prey, conspecific) and abiotic (turbulent flow, current) sources among hatchery-reared steelhead, in turn predicting reduced survival fitness after release. Physiological and behavioral studies are required to establish the functional significance of these morphological differences.

## Introduction

Salmon (*Oncorhyncus* spp. and *Salmo salar*) are central to the economies and cultural identities of many coastal communities in the Northern Hemisphere and a globally important food source [1]. Drastic declines in wild salmon populations across western North America over the past century attributable to a variety of anthropogenic factors have necessitated widespread supplementation of natural stocks with captively bred juveniles [1]-[4]. Five species of Pacific salmon and steelhead (*O. mykiss*) are reared *en masse* in federal, state and tribal hatcheries to provide for annual commercial and sport fisheries in the states of Alaska, Washington, Oregon, California, Idaho, and in British Columbia, Canada [4], [5]. Juvenile fish are typically reared in freshwater concrete tanks known as ‘raceways’ then released to out-migrate toward marine environments with wild conspecifics. An estimated 5 billion or more hatchery-reared juveniles are released into the North Pacific annually [6], with associated production costs in the hundreds of millions [7]. Unfortunately, despite augmented survival during the period of hatchery rearing, survival after release among hatchery-reared juveniles is typically low; hatchery adult return rates (i.e., smolt-to-adult survival rates) are commonly on the order of 1–2% or less (e.g., in chinook salmon, *O. tshawytscha*, and steelhead, [2], [8]). Wild adult returns, by comparison – though more variable and difficult to quantify precisely, requiring counts of both out-migrating juveniles and returning adults – can exceed 5–10% [2], [8], [9]. The average smolt-to-adult survival rate of wild-origin Queets River steelhead in Washington State over the period 1981–2007, for example, was 10.2% (Quinault Indian Nation, unpublished data).

Extensive measures have been undertaken to improve post-release survival of hatchery-reared juveniles. Such measures have included increased spill from hydroelectric impoundments during the spring months to ease juveniles' downstream migration [2], predator control efforts to reduce predation on outmigrating fish (e.g., by piscivorous birds and fish [10]), and ‘early intervention’ measures such as spawning of live-captured wild adults in the hatchery (known as ‘broodstocking,’ see [11]) or infrastructural alterations to hatchery facilities to mimic natural rearing conditions [12]. These and other ameliorative efforts have achieved a modicum of success. Returns of chinook and steelhead to the Columbia River Basin (Washington, Oregon, Idaho, and Montana), for example, have increased in recent years, anecdotally attributable in part to increased dam outflow during spring outmigration, though long-term effects have yet to be assessed [13]. A recent assessment of broodstocking programs, which use live-captured pre-spawn wild adults for hatchery production, demonstrated similar egg-to-adult survival rates (∼0.1%–0.6%) among progeny from two wild parents versus progeny from one wild and one hatchery parent, while the reproductive success (i.e., fecundity) of adults was found to decline rapidly (up to 40%) over successive generations of captive breeding, suggesting that both environmental and genetic factors may underlie survival deficits in hatchery-reared fish [11].

Chittenden et al. [14] recently examined the relative impacts of genetic and environmental factors (rearing conditions) on juvenile coho salmon (*O. kisutch*) development using a variety of morphological, physiological and behavioral assays. While essentially no differences were observed between genetic cohorts *within* rearing environments, numerous and in some cases drastic differences were found between groups reared in a standard hatchery versus a natural environment (river side channel with containment fences): Hatchery-reared fish, resultant of scheduled feeding, were larger than natural-reared fish, but exhibited comparatively high rates of fin damage, eye damage, scale loss and otolith crystallization – a condition associated with hearing deficits in hatchery-reared chinook salmon [15]. Moreover, natural-reared fish exhibited significantly greater swimming endurance and predator avoidance behavior than hatchery-reared fish. Finally, natural-reared fish migrated uniformly downstream after release, while hatchery-reared fish strayed both upstream and downstream. This assortment of attributes – particularly impoverished swimming, predator avoidance, and migratory behaviors – suggests significant impairment of motor and sensory systems in hatchery-reared fish. One sensory system particularly important to these and other survival-mediating behaviors, which has not been studied in the context of hatchery- versus wild survival fitness, is the lateral line mechanosensory system.

### Effects of hatchery rearing on the lateral line?

The lateral line is a sensory system possessed by bony and cartilaginous fishes. Lateral line end organs, known as neuromasts, are comprised of clusters of mechanoreceptive sensory hair cells. Lateral line hair cells detect low-frequency (DC-200 Hz) water motions caused by biotic and abiotic sources, contributing to many critical behaviors including prey capture, predator avoidance, schooling, orientation to currents, and communication (for reviews, see [16], [17]). In *Oncorhynchus*, the lateral line is additionally known to contribute to station holding in currents [18], prey tracking and capture [19], and spawning behaviors (in *O*. *nerka*, [20]). Most species, including *Oncorhynchus* spp., possess two major classes of neuromasts – canal neuromasts (CN), which are rooted inside subcutaneous canals on the animal's head and trunk, and superficial neuromasts (SN), which are rooted on the animal's skin or scales [21]–[23]. SN detect relatively low-frequency signals, such as stream flow and current wakes, while CN detect more rapid hydrodynamic fluctuations [24]. The relative abundance of each neuromast type is species- or population-specific and thought to depend on adaptation to the local hydrodynamic environment [25]–[27]. The hydrodynamic environment is a hallmark difference of hatchery and natural rearing conditions: flow-through systems in typical hatcheries are constant- and low-velocity, while natural stream flow is variable, turbulent, and generally higher-velocity. It is plausible that such differences could produce phenotypic plasticity in lateral line morphology, such as differences in neuromast number between hatchery-reared and wild (or ‘natural-reared’) fish.

Additionally, while CN are protected from direct contact with the external environment by skin or scales, SN protrude into the water immediately surrounding the fish. Thus, in high-density rearing environments typical of hatcheries, where a high degree of negative physical interaction occurs among juveniles – precisely the cause of fin deformations, scale loss, and other tissue damage [14], [28] – it is plausible that the SN of hatchery-reared juveniles could be damaged or outright ablated during the period of captivity. Neuromasts rooted on lost scales would certainly be ablated. Wild juveniles, which inhabit relatively open-field environments in much lower densities, should not be susceptible to the same degree of negative interaction and should thus possess comparatively intact SN.

The present study examined the hypothesis that hatchery-reared salmonids have fewer SN than their wild-origin conspecifics. Fluorescent imaging techniques were employed to assess lateral line morphology (neuromast number and distribution) and neuromast morphology (hair cell number) in individuals from two groups of hatchery-reared and one group of wild-origin juvenile steelhead. Fish were obtained from genetically similar stocks of steelhead native to Washington State's Olympic Peninsula (Queets-Quinault WRIA, [29]). Significantly fewer SN were observed in hatchery-reared juveniles, suggestive of functional deficits in lateral line-mediated behaviors, and perhaps reflective of behavioral deficits reported previously [14], [30]. In additional analyses, otolith composition and brain weight were also found to be different in hatchery-reared juveniles as compared to their wild-origin counterparts, in agreement with previous work in other *Oncorhynchus* species [12], [14], [31].

## Results

### Wild juveniles had significantly more SN than hatchery juveniles

**Figure 1:**
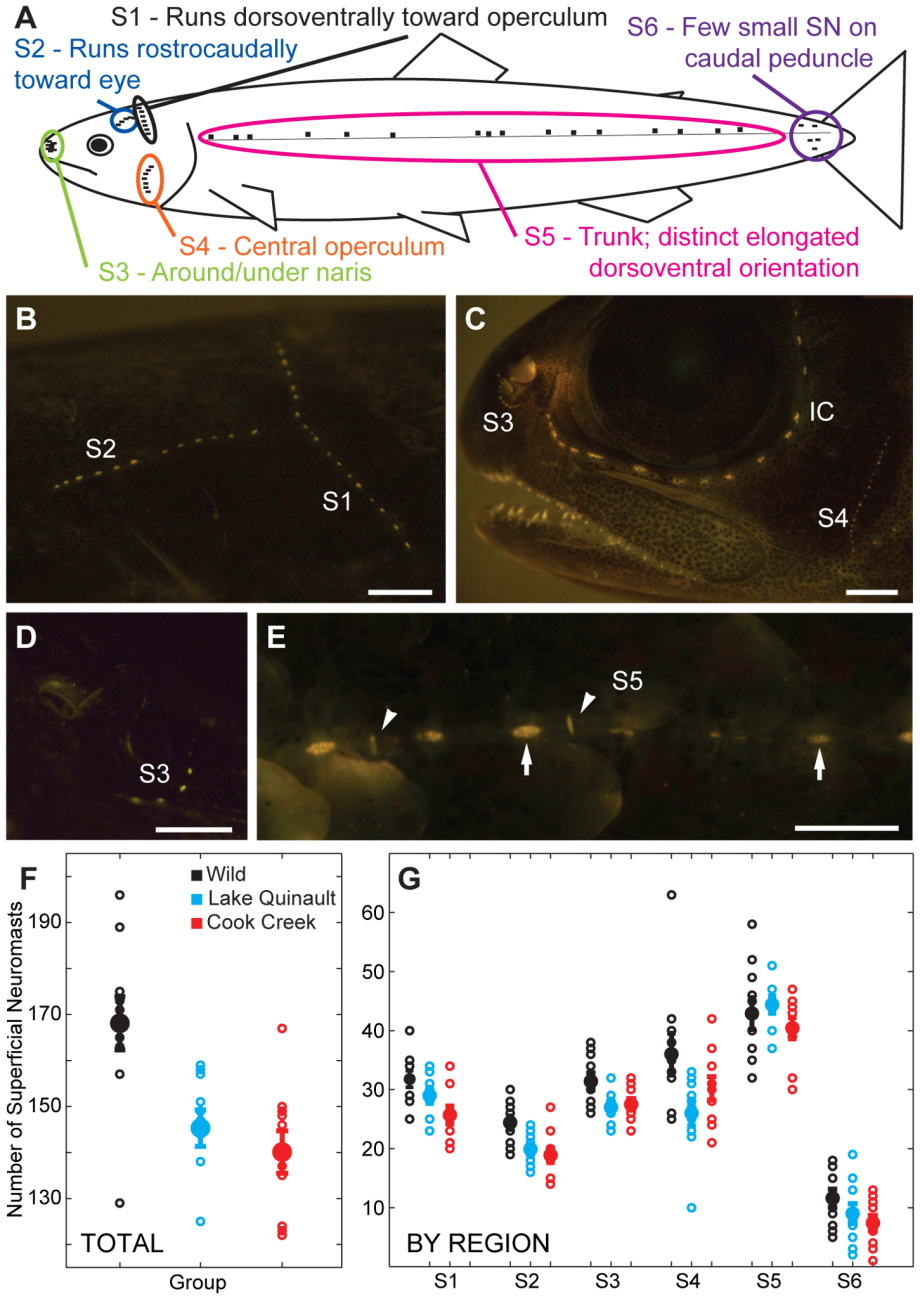
Differences in neuromast number across groups. (A) SN were clustered in one of six discrete ‘stitches’ or groupings, which we term for convenience in the present report S1–S6. Each of these stitches was treated as a region of interest for analysis of neuromast number. (B–E) Examples of DASPEI-labeled neuromasts from wild-origin juveniles (B–C) or Cook Creek hatchery fish (D–E). (B) Stitches S1–S2, showing how S2 intersects S1 near its midpoint, running anteroventrally toward the eye. Scale bar = 1 mm. (C) Low-magnification image of the left side of the head, showing the stitch around the naris (S3) and the stitch over the operculum (S4). The infraorbital canal (IC) is also labeled in this image. This canal was not clearly visible in all specimens so neuromast number was not quantified. Scale bar = 2 mm. (D) Stitch S3 (bordering a right-side naris) under higher magnification. Scale bar = 1 mm. (E) SN from stitch S5 (arrowheads), which sits atop the trunk canal (arrows). Trunk canal neuromasts are elongated in the rostrocaudal direction, while adjacent SN are oriented dorsoventrally. Scale bar = 1 mm. (F) Total neuromast number (summed across left and right sides) per fish (open circles) and per group (filled circles, mean±1 SEM, *n* = 10 fish per group). There were significant differences in neuromast number between groups (one-way ANOVA *F*_1,2_ = 9.45, *p* = 0.001). (G) SN number comparisons within each ROI using one-way ANOVA followed by Tukey's post-hoc analysis. Individual and group data are plotted across ROIs. Statistical tests are summarized are in Table 1.

The lateral line was visualized under a fluorescent dissecting microscope using the mitochondrial potentiometric vital dye DASPEI (2-(4-(dimethylamino)styryl)-N-Ethylpyridinium Iodide), which robustly labels lateral line hair cells (e.g., [23], [32], [33]; see Methods and Materials). SN were readily visible on all DASPEI-labeled juveniles. While the stereotyped morphology and distribution of CN in juvenile *O. mykiss* is well documented [34], [35], the less prevalent SN are less well described in the literature. SN were generally clustered in one of five or six discrete ‘stitches’ or groupings, which we term S1–S6 in the present report (see Fig. 1A). S1 is a highly stereotyped stitch of ∼12–20 neuromasts (per side) running dorsoventrally from the dorso-lateral surface of the head to the dorsal border of the operculum. S2, a smaller stitch of ∼6–10 neuromasts, intersects S1 near its midpoint, running anteroventrally toward the eye. Although S1 and S2 were often nearly continuous and thus might be considered a single stitch, S2 was occasionally discrete (removed anteriorly from S1 by a small distance), leading us to designate it separately (see Fig. 1B). S3 is another stereotyped stitch of ∼12–16 neuromasts that encircles the ventral ∼half of each naris, with 2–3 of the medial-most neuromasts located just lateral to the tip of the snout (Figs 1C, 1D). S4 is a somewhat variable ∼8–25 neuromast crescent-shaped stitch centered on the cartilaginous operculum (Fig. 1C). S5 is a more diffuse and irregular stitch of relatively large and elongate SN occurring along the length of the trunk; S5 neuromasts are located near the trunk CN, typically adjacent to canal pores in lateral line scales. Interestingly, individual S5 neuromasts tended to be oriented such that the long axis of the neuromast was orthogonal (dorsoventral orientation) to the characteristic rostrocaudal orientation of the simultaneously visible CN (see Fig. 1E). Finally, S6 was identified as an irregular grouping of SN on the caudal peduncle. S6 neuromasts varied in number from ∼0–10 and were conspicuous for their small size, caudal displacement from other SN on the trunk and irregular occurrence; some individuals possessed one or a few scattered S6 neuromasts, while others possessed several on each side, sometimes arranged linearly and extending nearly onto the caudal fin.

Figure 1F plots total neuromast number (summed across left and right sides) per fish (open circles) and per group (filled circles, mean ± 1 SEM, *n* = 10 fish per group). Data were analyzed by one-way ANOVA. The main effect of group membership was significant (*F_1,2_* = 9.45, *p* = 0.001), accounting for 41% of the variance in total SN number (*partial η^2^* = 0.41). Post-hoc pair-wise tests (Tukey's) demonstrated that wild juveniles (mean total SN  = 178) had significantly more SN than both Cook Creek (*mean* = 150) (*p* = 0.001) and Lake Quinault (*mean*  = 155) (*p* = 0.007) juveniles. To ascertain that differences in neuromast number were not related to differences in body size across groups, correlations between neuromast number and fork length (mean in *mm* ± SD: wild = 171.5±32.9, Cook Creek = 181.7±12.7, Lake Quinault = 185.1±16.5) or body weight (mean in *g*±SD: wild = 41.7±20.5, Cook Creek = 54.3±12.8, Lake Quinault = 61.3±17.7) were assessed. SN number bore no relationship to either measure (length, *R^2^* = 0.01, *p = *0.59; weight, *R^2^* = 0.01, *p = *0.61). Additionally, the average number of right-side trunk CN, which was assessed for each fish to ascertain effective DASPEI labeling, varied less than 2 neuromasts across groups (range = 114.5–116.4). Thus, SN number appeared to vary uniquely across groups.

To further investigate differences in lateral line morphology across groups, SN number was compared within each region of interest (ROI) using the same procedure applied above (one-way ANOVA followed by Tukey's pair-wise testing). Individual and group data are plotted across ROIs in Fig. 1G; statistical tests are summarized in Table 1. The significantly greater total number of SN in wild than hatchery juveniles was generally reflected within all ROIs, excepting S5 and S6, in which there were large within-group variances and no significant cross-group differences (*p*>0.05).

**Table 1:** Statistical comparison of SN number across groups.

	ANOVA	W vs. LQ	W vs. CC	LQ vs. CC
**S1**	**0.02**	0.36	**0.01**	0.25
**S2**	**<0.01**	**0.02**	**<0.01**	0.79
**S3**	**0.02**	**0.03**	**0.05**	0.94
**S4**	**0.04**	**0.03**	0.29	0.51
**S5**	0.39	.	.	.
**S6**	0.16	.	.	.

Observed *p*-values for ANOVAs and post-hoc pairwise tests (Tukey's) assessing cross-group differences in SN number by anatomical region (see Fig. 1A). Post-hoc tests were not conducted for S5 or S6, where the omnibus ANOVA demonstrated no main effect of group. Bolded values indicate significance at *p*<0.05.

### Hair cell number per neuromast did not differ across groups

**Figure 2:**
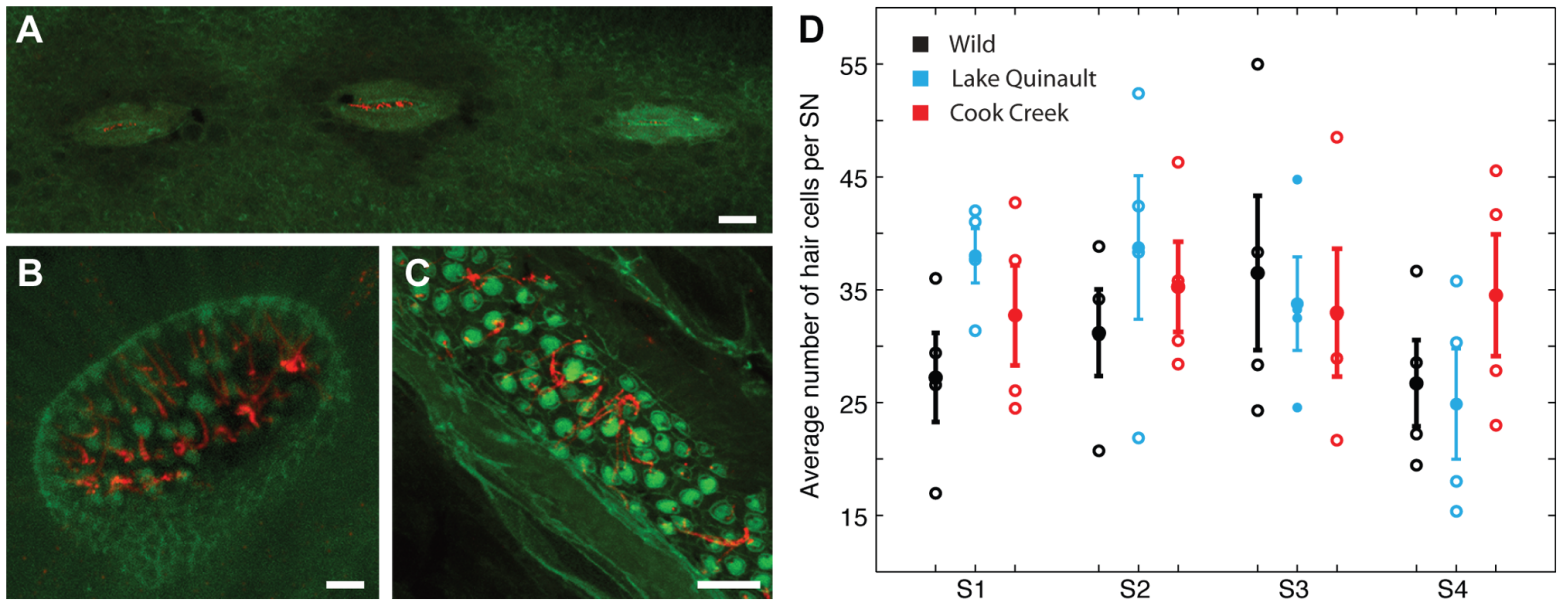
High variability in hair cell number within neuromasts. (A–C) Confocal images (brightest-point projections) of neuromasts double-labeled with anti-acetylated tubulin (red) and phalloidin (green), showing the kinocilia and hair bundles/cuticular plates, respectively. The phalloidin label also delineates overall neuromast architecture. (A) Three neuromasts from stitch S2 of a wild-origin fish, showing neuromast morphology and spacing. (B) Single SN from stitch S3 of a Lake Quinault hatchery fish, demonstrating the rounded morphology sometimes observed. In contrast, elongated SN were more typically noted, illustrated here by the S5 neuromast from a Cook Creek fish (C). (D) Average hair cell number for 4 randomly selected individuals (open circles) from each group for which complete data (SN in all four dissected ROIs S1–S4; range 3–21 SN per ROI) were available, and group means (filled circles, mean ± 1 SEM) for each ROI. There were no significant cross-group differences in hair cell number (*p*>0.05).

The most highly stereotyped SN stitches (S1–S4) were dissected off of 4–7 randomly selected fish in each group for post-fixation labeling (see Materials and Methods). Under high magnification, the shape of individual neuromasts was easily visualized, ranging from nearly round to ovoid or elongate (Fig. 2A–C). Tubulin-labeled lateral line nerve fibers were occasionally visible in the vicinity of hair cell bodies, but neither with sufficient resolution nor consistency to allow for systematic quantification and analysis. The number of tubulin or phalloidin/tubulin-labeled hair cells within each neuromast was highly variable within stitches, within individual fish, and within groups. Fig. 2D plots average hair cell number for 4 randomly selected individuals (open circles) from each group for which complete data (SN from all four dissected ROIs) were available, and group means (filled circles, mean ± 1 SEM) for each ROI. The number of SN in each stitch used to compute the average hair cell number depended on the ROI, ranging 3–21 SN for stitches S1–S4. Cross-group differences were assessed statistically by one-way ANOVA for each ROI. These tests revealed no significant cross-group differences in average hair cell number per neuromast within any ROI (*p>0.05*). We note, as an aside, that it was therefore not the case that hatchery-reared fish with fewer SN had somehow compensated for reduced neuromast number with increased hair cell density.

### Otolith crystallization in hatchery-origin juveniles

**Figure 3:**
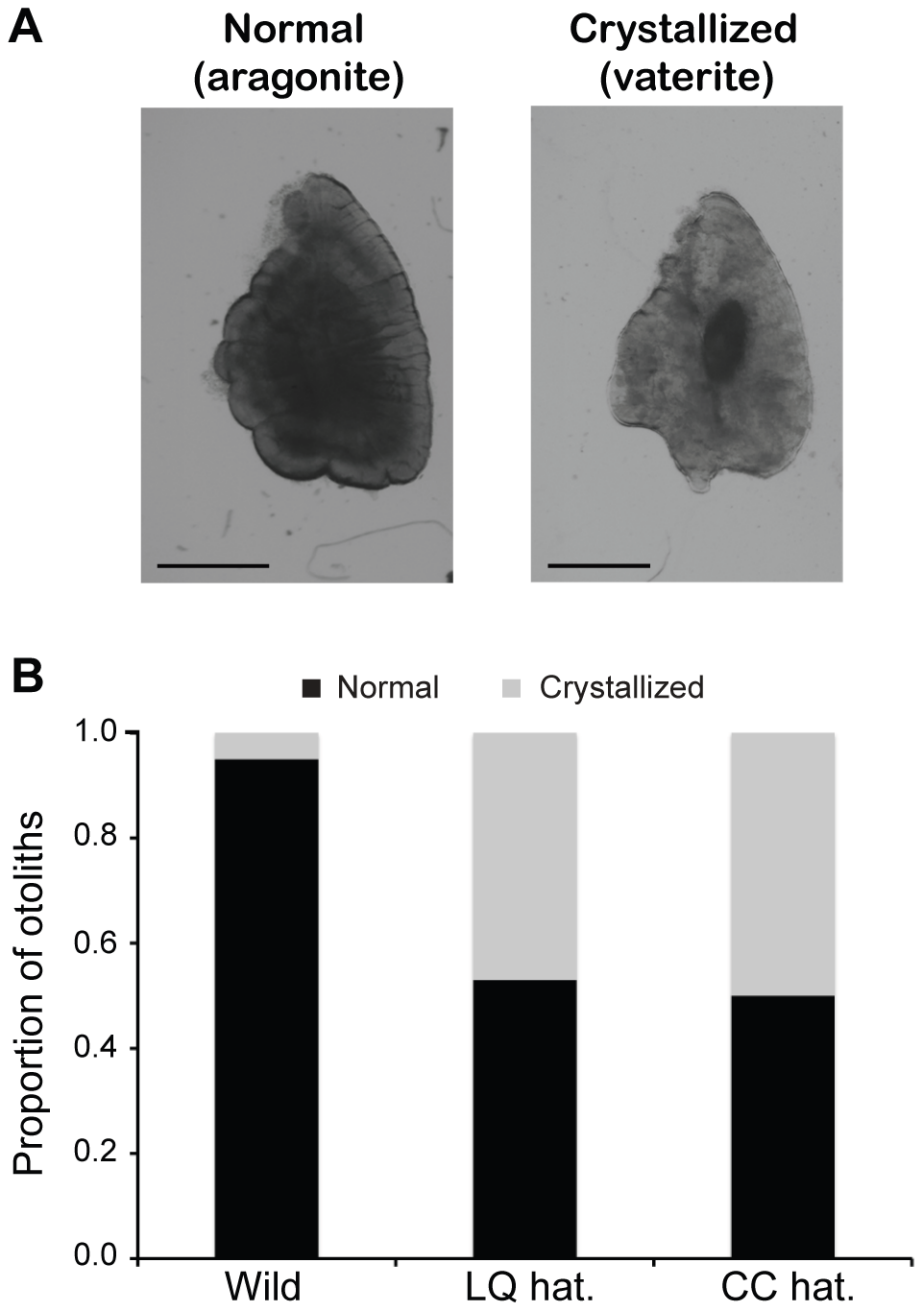
Sagittal otolith calcium carbonate composition. Transmitted light images of a normal (aragonite) sagitta from a wild-origin juvenile (left) and a crystallized (vaterite) sagitta from a Cook Creek hatchery fish (right). Scale bars in each image  =  1 mm. (B) Relative proportion of aragonite (black) and vaterite (gray) sagittae in fish from each population (wild *n* = 20 otoliths (10 fish×2 otoliths each), Cook Creek = 28 otoliths, Lake Quinault = 34 otoliths). Both groups of hatchery fish had a significantly higher proportion of crystallized otoliths than wild fish (Chi-square tests, *p* = 0.001).

As noted in the Introduction, otolith crystallization (characterized by deposition of the calcium carbonate polymorph *vaterite* in place of *aragonite*), has been observed in hatchery-reared chinook and coho salmon, and specifically associated with reduced auditory sensitivity in chinook [15], [31], [36]. To examine otolith composition in *O. mykiss*, saccular otoliths (sagittae) were dissected from all collected specimens in all three groups (wild *n* = 20 otoliths (10 fish×2 otoliths each), Cook Creek = 28 otoliths, Lake Quinault = 34 otoliths) and classified as either normal (aragonite) or crystallized (vaterite) by observing the relative opacity of the otolith using a stereomicroscope and transmitted light (see also Materials and Methods). Normal otoliths appear as smooth, opaque structures, while otoliths containing significant vaterite deposition are translucent with irregular surface contours (Fig. 3A). Wild-origin juveniles had primarily aragonite-containing otoliths (95%, Fig. 3B). In contrast, hatchery-reared fish had approximately 50% crystallized otoliths (Lake Quinault, 47%, Cook Creek, 50%). Chi-square analyses determined that the difference between wild fish and both groups of hatchery fish was significant (wild vs. Lake Quinault, *χ^2^* = 10.33, *p* = 0.001, wild vs. Cook Creek, *χ^2^* = 11.00, *p* = 0.001), while the difference between groups of hatchery fish was not (*p*>0.05).

### Reduced brain weight in hatchery-origin juveniles

**Figure 4:**
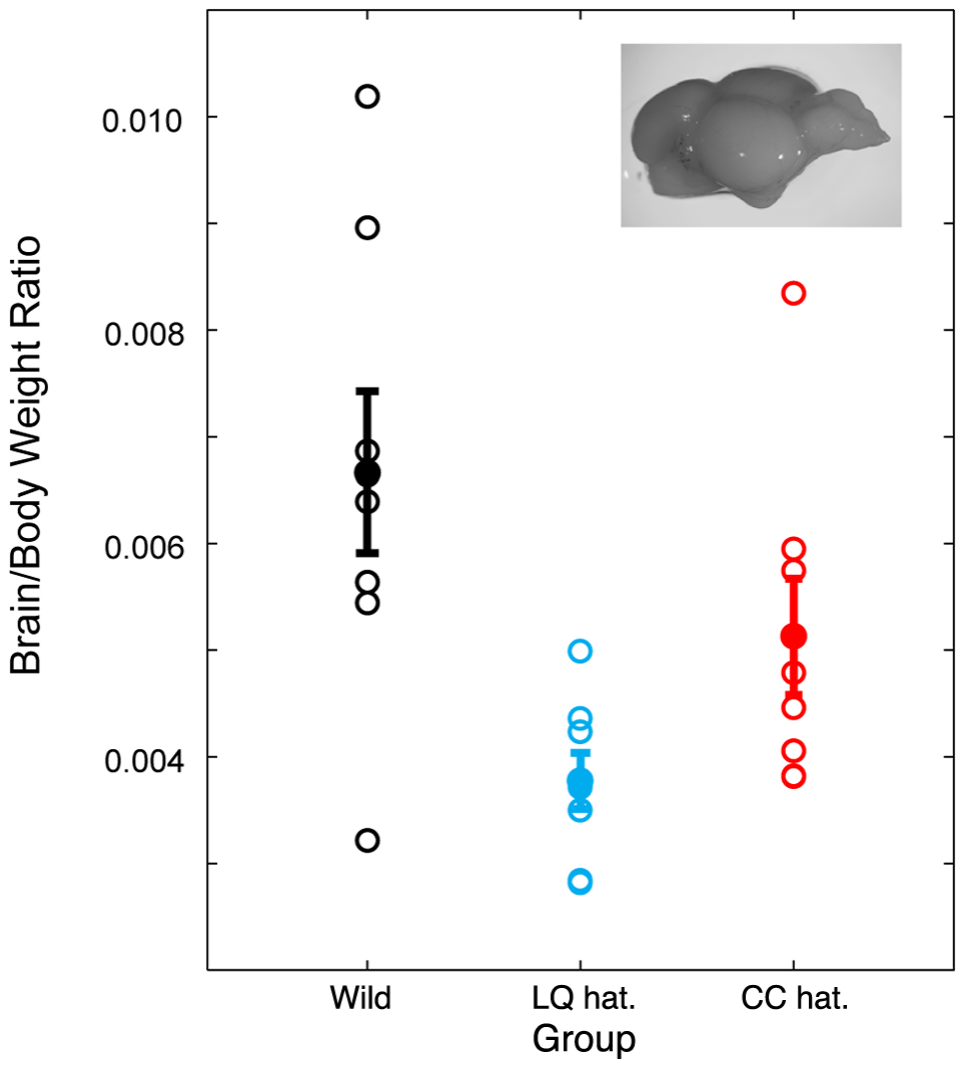
Brain weight differs between groups. Normalized brain weight, expressed as the brain/body weight ratio, of 8 randomly selected individuals (open circles) from each group and means for each group (filled circles, mean ± 1 SEM). The inset shows a brain from a wild-origin juvenile with the olfactory bulbs removed. Scale bar = 2 mm.

In a final analysis, following on the work of Kihslinger and colleagues [12], [37], brain weights were determined for 8 fish from each group. As the olfactory bulb was damaged in some animals, weights for all brains were taken after olfactory bulb removal (see Figure 4 inset). Data are presented as brain weight normalized to body weight. We note that brain weight was measured post-fixation, while body weight was measured prior to fixation (immediately following sacrifice). Thus, particularly with the removal of olfactory bulbs, obtained brain weights underestimate the live brain weight in all fish; nonetheless, fish from all groups were processed identically. As seen in Fig. 4, normalized brain weight differed significantly across groups (*F*_1,2_ = 6.68, *p = *0.006). Follow-up pair-wise tests indicated that brain-to-body weight ratio in wild juveniles (*mean* = 0.0067) was significantly greater than in fish from Lake Quinault (*mean* = 0.0038; *p* = 0.004), but not Cook Creek fish (*mean = *0.0051; *p* = 0.15). Lake Quinault and Cook Creek normalized brain weights were not significantly different (*p* = 0.23).

## Discussion

Salmon are a globally important food source and a staple of coastal economies in western North America, Asia, and Europe [1]. Anthropogenic factors including habitat degradation, hydroelectric development, and sustained high levels of harvest have led to global declines in natural populations, and in some cases local extinctions [2], [3], [38]. Although hatchery-rearing programs enable high-volume production of juveniles, offering to mitigate losses in natural production, hatchery programs are perpetually high-cost and tend to produce fish that survive at low rates [2], [5], [38]. While restoring historic salmon habitat to promote natural recovery of wild populations represents a promising solution in some cases (e.g., Washington State's Elwah River, [39]), identifying factors limiting the survival of hatchery-reared fish is paramount to the future of most salmon fisheries. In the foregoing sections we have demonstrated several significant morphological differences between hatchery-reared juvenile *O. mykiss* and wild juveniles from a nearby stream: Hatchery-reared fish possessed significantly fewer superficial lateral line neuromasts, a much higher proportion of crystallized otoliths, and relatively smaller brains. This is the first report, to our knowledge, of lateral line differences between captively reared and wild-origin fish. Taken together, these observations suggest deficits in mechanosensory function in hatchery-reared fish that may negatively impact survival fitness after release into the wild. In the remaining sections we consider the functional implications of these data and limitations in their interpretation.

### Functional role of the lateral line system in salmonids

Among other teleosts, lateral line function in *Oncorhynchus* has been particularly well-characterized [18]–[20], [22], [33], [34], [40]. While a majority of studies have focused on CN, which are larger than SN and respond to a broader range of input frequencies [41], SN have been shown to mediate several behaviors critical to survival. In a series of experiments by Montgomery and colleagues, chemical ablation of SN in *O. mykiss* with gentamicin was shown to significantly reduce or abolish flow orientation (rheotaxis), station holding in currents, and prey tracking and capture [18], [19], [22] (although the SN-specificity of gentamicin toxicity is questionable; see [23] for a discussion of caveats toward the use of aminogylcosides in lateral line behavioral studies). The specific role of SN has not been studied in other lateral line-mediated behaviors, but pharmacological blockade of both SN and CN with cobalt chloride abolished spawning behavior in *O. nerka* [20]. In many teleosts the lateral line is also known to contribute to predator avoidance (for review, see [16], [17]). These data suggest that hatchery-reared juveniles with reduced SN might be less sensitive than wild fish to biologically relevant biotic and abiotic stimuli (e.g., in-stream flow obstacles, prey, or predators) during the period of outmigration and perhaps beyond. While this possibility seems particularly tenable in light of recent studies demonstrating reduced predator avoidance and altered swimming and migration behaviors in hatchery-reared *Oncorhynchus* [14] and reduced foraging success in hatchery-reared *S. salar* [30], carefully designed behavioral and physiological assays are required to validate such conjecture.

### Ecological significance of otolith crystallization

Hair cells of the saccule, the primary hearing organ in salmonids and most other fishes, are acoustically stimulated via their motion relative to the adjacent saccular otolith (sagitta). Vibration of the high-density sagitta in response to sound lags behind that of the saccular epithelium and associated hair cells, leading to rapid deflections of hair cell stereocilia and, ultimately, transduction of the acoustic signal. Correspondingly, otolith crystallization – deposition of *lower-density* vaterite in place of high-density aragonite - has been associated with reduced auditory sensitivity (i.e., poorer hearing) in hatchery-reared chinook salmon [15]. In the present study, fish from both Lake Quinault and Cook Creek hatcheries had high proportions of vaterite sagittae as compared to wild-origin juveniles, suggesting likely auditory deficits in both groups of hatchery fish. Physiological and/or behavioral testing of auditory sensitivity is necessary to fully explore this possibility. It is interesting that the Lake Quinault fish possessed a slightly higher proportion of aragonite sagittae than the Cook Creek fish, mirroring the trend in SN number seen in these hatchery populations (i.e., more SN in Lake Quinault juveniles).

### Reduced brain size in hatchery-reared fish

Kihslinger et al. [12] previously demonstrated that the average brain volume (olfactory bulb and telencephalon) of wild chinook salmon juveniles was greater than that of hatchery-reared juveniles, while the brain volumes of juvenile chinook from a ‘Natural Rearing Enhancement System’ (NATURES) hatchery and a standard hatchery were not significantly different. Our analysis of brain weight in *O. mykiss* reflected the same pattern in that the normalized brain weight in wild fish was greater than that of fish from both a standard hatchery (Cook Creek, a raceway facility) and a more ‘natural’ hatchery (Lake Quinault, a net pen facility on a natural lake), although the difference was only statistically significant for the Lake Quinault group. There was no significant difference between the two hatchery groups. Future studies should evaluate the detailed brain morphology of hatchery- versus wild-origin juveniles; in addition to other previously described differences (e.g., reduced olfactory bulb volume in hatchery fish, [12]), the present results suggest possible atrophy of brain areas devoted to processing mechanosensory information (i.e., lateral line and auditory nuclei) in hatchery fish.

### Limitations on the interpretation of presented data and future directions

The present study was a purely structural assessment of mechanosensory anatomy and gross brain size in fish from three different rearing environments. Although the stocks of fish included in the study were selected for their geographic proximity and genetic similarity, the stocks are not genetically identical [29]. Given the basic physiological similarity among species of *Oncorhynchus* [42] and among stocks of *O*. *mykiss* specifically, it seems highly unlikely that the observed differences in lateral line, otolith, and brain characteristics would manifest simultaneously as a result of purely genetic (rather than environmental) differences (cf. [14]), but this possibility cannot be completely excluded without replicating the study in individuals from common parentage. Additional studies using more animals from a variety of stocks and other members of the *Oncorhynchus* genus (and perhaps also *S. salar*) will also work to establish the generalizability and prevalence of the morphological differences we observed.

Finally, we reiterate that behavioral and neurophysiological studies are required to evaluate the functional significance of our anatomical observations. In the fish auditory system, damage-induced loss of hair cells is correlated with decreased sensitivity to sound (i.e., increased thresholds), demonstrating a strong relationship between hair cell number and functional sensitivity [43], [44]. A recent study by Suli and colleagues also demonstrated a systematic relationship between lateral line hair cell loss and flow orientation behavior in larval zebrafish [45]; whether such a structure-function relationship exists for the lateral line (and SN specifically) of *O. mykiss* remains to be determined. Longitudinal studies are also indicated in order to establish the time course and proximal causes of changes in neuromast number within the hatchery environment. Experiments explicitly designed to assess the relative contributions of the hydrodynamic environment versus negative conspecific interactions (i.e., acute ablation) to observed differences in neuromast number will work to parse the factors affecting lateral line development. Given that fish possess the ability to regenerate neuromasts [32], [46], [47], it seems unlikely that reduced SN number in hatchery fish would be purely attributable to acute trauma and tissue damage, although the time course of regeneration is unknown for juvenile salmonids and scale loss would preclude rapid regeneration of any neuromasts rooted at the site of the lesion. While the future of a species that undergoes transoceanic migrations necessarily depends on a host of local and global factors, identification of factors limiting survival at the level of the individual may elucidate avenues to ameliorative solutions in the future, such as rearing practices that promote normal lateral line development (e.g., more natural hydrodynamic conditions combined with lower rearing densities).

## Methods

### Ethics Statement

All procedures were approved by the Institutional Animal Care and Use Committee at Washington State University, protocol number 04237-001. All animals were collected in cooperation with and under permit by Quinault Indian Nation's Department of Natural Resources.

### Animals

Juvenile steelhead (*Oncorhynchus mykiss*) were obtained from Washington State's Olympic Peninsula in spring of 2012. Wild-origin juveniles (*n* = 10) were collected from a smolt trap operated by Quinault Indian Nation on Mud Creek, a tributary of the lower Queets River near Queets, WA. Hatchery-reared juveniles were obtained from Cook Creek National Fish Hatchery (*n* = 14) – a facility in which juveniles are reared in traditional concrete raceways – and from Lake Quinault Hatchery (*n* = 17) – a facility operated by Quinault Indian Nation in which juveniles are reared in suspended net pens on a large natural lake. Collected fish were placed in 25-gallon aerated tanks filled with cool (10–12 °C) water from their respective environments. Live fish were transported immediately to Washington State University in Vancouver, WA, and held in an isolated room for no more than 48 h prior to the commencement of lateral line labeling and other experimental procedures.

### Vital dye labeling and neuromast counts

Fish were lightly anesthetized in a bath of buffered MS-222 (tricane methanesulfonate, Western Chemical, Inc.) until righting behavior was visibly reduced. Fish were then placed for 20 min in a 0.005% solution of the fluorescent vital dye DASPEI ((2-(4-(dimethylamino)styryl)-N-Ethylpyridinium Iodide); Invitrogen), a mitochondrial potentiometric dye that robustly labels lateral line hair cells (e.g., [23], [32], [33]). Fish were then rinsed in fresh water and anesthetized more deeply with MS-222 until opercular movement slowed. MS-222 concentrations were adjusted for each animal to obtain a satisfactory level of anesthesia; fish were not restrained, and sudden movements would have been detrimental to the accuracy of neuromast counts. All solutions were changed frequently and held at a temperature of 10–12 °C to avoid thermal shock. DASPEI-labeled neuromasts were observed on a Leica M165 FC stereomicroscope equipped for epifluorescence and images were captured with a Leica DFC 450C CCD-cooled camera. All visible SN were counted on all fish. A highly stereotyped subset of CN (right trunk canal) was also counted on each fish to verify the efficacy of labeling. SN were generally found in stereotyped stitches, classifiable as six discrete regions of interest (ROIs) on each side of the body (see Fig. 1). After neuromast quantification, fish were euthanized with an overdose of buffered MS-222, measured (fork length), weighed, and fixed in 4% paraformaldehyde (Sigma) in phosphate-buffered saline (PBS) at 4 °C for approximately 48 hours. Fish were then rinsed twice in PBS and ROIs were dissected off for post-fixation labeling (Fig. 1; see below).

### Post-fixation labeling and hair cell counts

Four ROIs – stitches of SN occurring in stereotyped patterns and locations on the dorso-lateral surface of the head (S1, S2), under the nares (S3), and on the operculum (S4) were dissected off the right side of each fish for further processing. Tissue was labeled with an antibody to tubulin to visualize kinocilia and cell bodies and with phalloidin to visualize stereocilia [14], [31], [48]. All steps were performed in PBS (pH 7.4) at room temperature unless otherwise noted. Tissue samples were rinsed in fresh PBS, then in double-distilled water for 20–30 minutes to facilitate tissue clearing and improve antibody penetration. Tissue was then blocked in 5% normal goat serum (Sigma) in PBS supplemented with 1% Triton-X (PBST) (Sigma), and incubated overnight at 4 °C in PBS with 1% normal goat serum, 1% Triton-X, and mouse monoclonal acetylated tubulin antibody diluted 1∶2500 (Sigma). Tissue was rinsed in PBST and incubated for 2–4 hours in Alex Fluor 568 goat anti-mouse secondary antibody (Invitrogen) diluted 1∶500 in PBST, rinsed in fresh PBST, and incubated in proteinase K (80 µg/ml) for 1 hr at 37 °C. Tissue was then incubated overnight at 4 °C in Oregon Green phalloidin (Invitrogen) diluted 1∶100 in PBST. Tissue was rinsed in fresh PBST, then in PBS, and stored in 1∶1 PBS:glycerol for imaging. Neuromast imaging was performed on a Leica DMI 4000 B compound epifluorescent microscope equipped with a Leica DFC 420C camera, or on an Olympus FV1000 confocal microscope with associated Fluoview software. Images were collected as either single planes of section, or as z-series when the entire neuromast could not be focused into a single plane. Maximum point projections were produced with Image Analyzer Pro (for Leica images) or with ImageJ (for Olympus images). Hair cells were counted in each neuromast using ImageJ with the Cell Counter plug-in. Only tubulin-labeled cells were counted because the tubulin label was robust while the phalloidin labeling was more variable.

### Otolith analysis

Saccular otoliths (sagittae) were dissected from the braincase, rinsed in distilled H_2_O, and examined with a Leica M165 FC stereomicroscope using both transmitted and reflected light. Normal (aragonite-containing) sagittae appear opaque, while those with a substantial proportion of vaterite (‘crystallized’ otoliths) are translucent. Otoliths composed of ≥ 33% vaterite were classified as crystallized, those with less vaterite (or those completely aragonite) were classified as normal [15], [49].

### Brain weight analysis

Following on work of Kihslinger and colleagues [12], [37], we dissected brains from hatchery and wild juveniles to assess whether (1) wild juveniles, which inhabit a markedly more diverse environment than hatchery juveniles might possess larger brains than hatchery juveniles and (2) whether fish from the Lake Quinault hatchery, which are reared in net pens suspended on a natural lake – a more naturalistic environment than the concrete raceway of fish from the Cook Creek hatchery – might also exhibit larger brains than the Cook Creek juveniles. Brains were dissected from 8 fish from each group. Because the olfactory bulbs were partially detached in a number of specimens, all brains were analyzed with the olfactory bulb removed. Brains were weighed and the normalized brain weight was computed by dividing the obtained value by the body weight of the fish (cf. [12]). Since fish were weighed prior to fixation and brains were removed post-fixation, obtained values underestimate the live brain weight; nonetheless, the same procedure was applied to all fish, and cross-group comparisons should be unaffected.

### Statistical analysis

Cross-group differences in SN number were assessed using standard null hypothesis testing. Total (whole-fish) SN number and SN per ROI (see Fig. 1) were compared across groups by ANOVA. In cases where the omnibus ANOVA indicated a significant main effect of group, follow-up pairwise tests (Tukey's) were also conducted to assess the interrelationships among groups. Cross-group differences in hair cell number were assessed by computing for each fish the average number of hair cells per neuromast in each of 4 SN stitches (S1, S2, S3, S4) and conducting ANOVAs on these averages (main factor of group, one ANOVA per stich). As ANOVA yielded no significant omnibus main effects in any case and high variability within individual fish and within groups was clear (Fig. 2D), follow-up pairwise tests were not conducted in any case. Differences in the proportion of normal vs. crystallized otoliths between groups were analyzed using a chi-square test. Lastly, normalized brain weights were compared across groups by a one-way ANOVA, with follow-up pairwise tests (Tukey's).
